# Resetting *FLOWERING LOCUS C* Expression After Vernalization Is Just Activation in the Early Embryo by a Different Name

**DOI:** 10.3389/fpls.2020.620155

**Published:** 2021-01-13

**Authors:** E. Jean Finnegan, Masumi Robertson, Chris A. Helliwell

**Affiliations:** Commonwealth Scientific and Industrial Research Organisation (CSIRO) Agriculture and Food, Canberra, ACT, Australia

**Keywords:** vernalized state, epigenetic memory, histone H3, chromatin, gametogenesis

## Abstract

The reproductive success of many plants depends on their capacity to respond appropriately to their environment. One environmental cue that triggers flowering is the extended cold of winter, which promotes the transition from vegetative to reproductive growth in a response known as vernalization. In annual plants of the *Brassicaceae*, the floral repressor, *FLOWERING LOCUS C* (*FLC*), is downregulated by exposure to low temperatures. Repression is initiated during winter cold and then maintained as the temperature rises, allowing plants to complete their life cycle during spring and summer. The two stages of *FLC* repression, initiation and maintenance, are distinguished by different chromatin states at the *FLC* locus. Initiation involves the removal of active chromatin marks and the deposition of the repressive mark H3K27me3 over a few nucleosomes in the initiation zone, also known as the nucleation region. H3K27me3 then spreads to cover the entire locus, in a replication dependent manner, to maintain *FLC* repression. *FLC* is released from repression in the next generation, allowing progeny of a vernalized plant to respond to winter. Activation of *FLC* in this generation has been termed resetting to denote the restoration of the pre-vernalized state in the progeny of a vernalized plant. It has been assumed that resetting must differ from the activation of *FLC* expression in progeny of plants that have not experienced winter cold. Considering that there is now strong evidence indicating that chromatin undergoes major modifications during both male and female gametogenesis, it is time to challenge this assumption.

## Introduction

Plants must respond to environmental challenges to ensure their survival or reproductive success by changing patterns of gene expression. Gene expression is modulated not only by transcription factors but also by the associated chromatin environment, which can influence expression long after the event that triggered changes in local chromatin. Vernalization, the promotion of flowering in response to the prolonged cold of winter, is a well-studied example of the long-term memory provided by the epigenome (reviewed in Berry and Dean, 2015). In annual plants, the vernalized state is established during winter, maintained through the life of the vernalized plant but is reset in the progeny so that each generation can respond appropriately to the seasonal cycles (Lang, 1965). The timing of resetting differs in perennials in which vegetative growth is restored in some shoots on return to warmer conditions (reviewed in Turck and Coupland, 2014).

In *Arabidopsis thaliana*, the key gene in the vernalization response is the repressor of flowering, *FLOWERING LOCUS C* (*FLC*), which is downregulated by low temperatures. Initially repression is transient, but *FLC* repression is stabilized as the duration of the exposure to low temperature increases (Angel et al., 2011). As ambient temperatures rise at the end of winter, *FLC* repression is maintained allowing the induction of two promoters of flowering, *FLOWERING LOCUS T* (*FT*) and *SUPPRESSOR OF CONSTANS 1* (*SOC1*), and the subsequent transition to flowering (Helliwell et al., 2006; Searle et al., 2006). *FLC* is released from repression in the next generation allowing the progeny of a vernalized plant to respond to winter (Sheldon et al., 2000). Activation of *FLC* in the next generation has been termed resetting to denote restoration of the non-vernalized state in the progeny of a vernalized plant. The process of resetting the vernalized state has long intrigued biologists because tissues giving rise to male and female gametes are not set aside early in embryo development as they are in mammals, but rather develop from somatic tissue that has accumulated changes to the epigenome during vegetative growth (reviewed in Gehring, 2019).

The molecular events associated with the long-term repression of *FLC* have been elucidated. In contrast to the gradual decline in the abundance of *FLC* mRNA that occurs during an extended period at low temperatures, the downregulation of transcription occurs more rapidly, and this may be initiated by a cold-induced physical change in *FLC* chromatin (Csorba et al., 2014; Finnegan, 2015; Helliwell et al., 2015; Rosa et al., 2016). Cold exposure also results in the induction of antisense transcripts, collectively known as *COOLAIR*, as well as *VERNALIZATION INSENSITIVE 3* (*VIN3*), a PHD domain protein that associates with a vernalization-specific polycomb repression complex, Polycomb Repressive Complex 2 (PRC2; Sung and Amasino, 2004; de Lucia et al., 2008; Swiezewski et al., 2009). Initially repression of *FLC* is transient but becomes stabilized in a time dependent manner that is cell (and locus) autonomous (Angel et al., 2011; Berry et al., 2015; Yang et al., 2017). This switch is associated with stabilization of the nucleosome in the +1 position relative to transcription, loss of active chromatin marks, and gain of H3K27me3 within the nucleation region of *FLC* chromatin that encompasses the +1 nucleosome (Finnegan and Dennis, 2007; Angel et al., 2011; Finnegan, 2015). After winter ends, H3K27me3 then spreads across the entire locus in a DNA replication-dependent process (Finnegan and Dennis, 2007; Angel et al., 2011; Hyun et al., 2013; Finnegan, 2015; Yang et al., 2017). PRC2 activity is essential for the switch between transient and stable repression, suggesting that H3K27me3 accumulation is important for stable repression (Gendall et al., 2001; Helliwell et al., 2011; Yang et al., 2017). Recruitment of PRC2 to *FLC* chromatin is facilitated by the binding of the B3 transcriptional repressors, VAL1 and/or VAL2, to RY-1 and RY-2 motifs (TGCATG; R, purine, Y, pyrimidine; Swaminathan et al., 2008) that constitute a cold memory element (CME) within the nucleation region of *FLC*, during cold exposure (Questa et al., 2016; Yuan et al., 2016). VAL1 interacts directly with components of PRC1 and PRC2 (AtBMI1 and LHP1, respectively), and SAP18, part of the SIN3-histone deactylase complex that in turn binds the histone deactylase HDA19, to shut down transcription and indirectly recruit PRC2 (Questa et al., 2016; Yuan et al., 2016). It has long been thought that resetting reverses these changes by the removal of H3K27me3 and activation of *FLC* transcription. This could occur prior to, or during, gamete formation in the vernalized plant or post-fertilization, in the developing embryo. To determine whether resetting is a process unique to vernalized plants, we must first consider the timing and genetic requirements for expression of *FLC* in each generation of non-vernalized plants.

## *FLC* Expression Must be Activated in the Embryos of Non-Vernalized Plants

**Figure 1:**
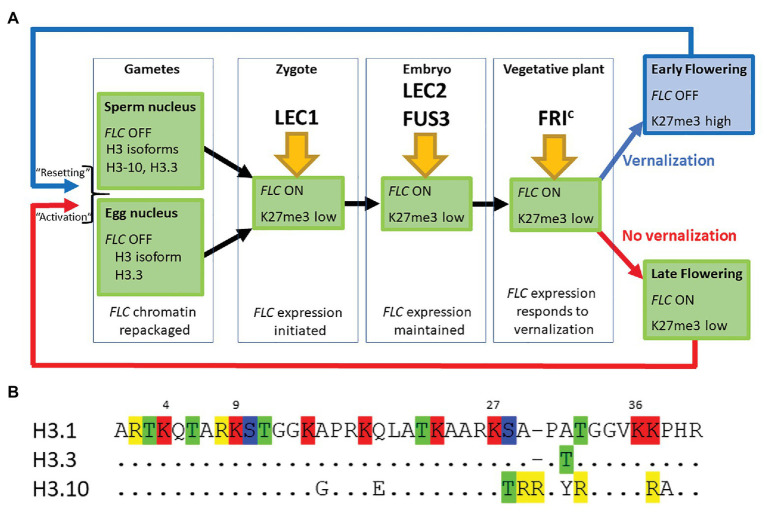
**(A)** *FLOWERING LOCUS C* (*FLC*) expression is established in the zygote and maintained through the life of the plant unless the plant becomes vernalized by exposure to prolonged cold during winter. *FLC* is inactive in mature gametes of both vernalized and non-vernalized plants where the chromatin is repackaged and epigenetic marks are lost. The major transcription factors determining *FLC* expression at each developmental stage are indicated. **(B)** The first 40 amino acids of three different isoforms of Histone H3 are shown. Single letter nomenclature is used and the dots shown in H3.3 and H3.10 indicate that the sequence is conserved with H3.1. The commonly methylated lysines are numbered above the sequence. Amino acids that can be modified are highlighted; R, me; T, ph; K, ac/me; S, ph. H3.10 and H3.3 each have the potential for novel modifications not seen in H3.1 and the substitution S-T28 (H3.10) may disrupt the activity of the kinase that normally phosphorylates that position. The changes adjacent to K27 may also prevent methylation of that residue. H3.1 denoted the protein encoded by *HTR3*, H3.10 by *HTR10*, and H3.3 by *HTR5*.

An *FLC::GUS* reporter construct, which mirrors the endogenous *FLC* gene, is expressed throughout the somatic tissues of non-vernalized plants and in both the carpel and stamens, but there is no expression in either the developing gametophytic embryo sac or in mature pollen (Figure 1A; Sheldon et al., 2008; Choi et al., 2009). Thus, even in the absence of vernalization, *FLC* is repressed during gametogenesis and must, therefore, be activated in the next generation. Activation of *FLC::GUS* is initiated at the earliest stage of embryo development although some β-glucuronidase (GUS) activity may result from maternal mRNA inherited *via* the egg cell (Sheldon et al., 2008; Tao et al., 2017; Luo et al., 2020). *FLC::GUS* expression continues to increase until early heart stage and is then maintained for the duration of embryo development (Figure 1A; Sheldon et al., 2008; Tao et al., 2017; Luo et al., 2020). In somatic tissue, a complex of proteins, the FRI complex (FRI^c^), which includes FRI, FRL1, FES, FLX, and SUF4, is required for expression of *FLC* (Choi et al., 2011). However, *FLC::GUS* is activated in young embryos in both *fri* and *suf4* mutants, suggesting that FRI^c^ is not essential for activation of *FLC* in the early stages, although it promotes *FLC* expression in globular embryos and throughout the later stages of embryo development (Choi et al., 2009; Tao et al., 2019). This raises the question of what initiates *FLC* expression in the zygote.

As *FLC* expression is observed as early as 1 day after pollination (DAP), it was proposed that activation of *FLC* would most likely involve the action of an embryo transcription factor (Tao et al., 2017). *LEAFY COTYLEDON 1* (*LEC1*), which is expressed in the zygote and throughout embryogenesis, is a component of a seed specific NF-Y (nuclear factor of the Y box) transcription factor that is a master regulator of embryo development (Lotan et al., 1998; Le et al., 2010). NF-Y transcription factors are conserved across kingdoms and have been shown to act as pioneer transcription factors that can bind their target recognition site in the context of nonmodified chromatin or even closed chromatin marked with the repressive modification, H3K27me3 (Fleming et al., 2013). They are comprised of three subunits, NF-YA, a site-specific DNA binding protein that targets CCAAT motifs and two proteins with a histone-domain fold, NF-YB and NF-YC that are structurally related to Histone H2B and H2A, respectively (reviewed in Mantovani, 1999).

To investigate the role of NF-Y on *FLC* activation, a null mutant of lec1, one of 10 NF-YB subunits encoded by the *Arabidopsis* genome, was introduced into Col^FRIS*f2*^. Loss of LEC1 activity partially suppresses the late flowering phenotype in non-vernalized plants, and the expression of both *FLC::GUS* and endogenous *FLC* is reduced throughout embryo development. Once established, the level of *FLC* expression appears to be fixed as the proportion of embryos showing weak, intermediate, or strong expression remains consistent across the first 3 DAP (Table 1A; Tao et al., 2017). *FLC* expression is even lower in embryos where there was decreased activity of all five members of the *LEC1* clade, indicating that NF-YB subunits from this clade act redundantly to activate the level of *FLC* in the zygote (Tao et al., 2017). Several lines of evidence indicate that LEC1 directly activates *FLC*. Mutation of the four putative NF-Y binding sites in the *FLC* promoter leads to early flowering; LEC1::FLAG is enriched at this region of the *FLC* promoter; *FLC* expression is induced in response to ectopic induction of a *LEC1* transgene and this is associated with the establishment of active chromatin at *FLC* (Tao et al., 2017).

**Table 1:** GUS expression during embryo development is determined by the activity of LEC1 in the zygote **(A)** or FUS3 activity in the embryo (**B;** adapted from Tao et al., 2017, 2019).

**(A)**				
**Genotype**	***LEC1 FLC::GUS***	***lec1 FLC::GUS***		
Phenotype	Very strong	Weak	Intermediate	Strong – very strong
1 DAP	96%	28%	49%	23%
2 DAP	98%	29%	49%	22%
3 DAP	98%	29%	48%	23%
**(B)**				
**Genotype**	***FUS3 FLC::GUS***	***fus3 FLC::GUS***		
Phenotype	Very strong	Weak	Intermediate	Strong – very strong
3 DAP	97%	12%	55%	32%

While LEC1 is key to the activation of *FLC* in the zygote, two other embryo-specific transcription factors, LEC2 and FUSCA 3 (FUS3), are needed to maintain *FLC* expression from 2 DAP (Figure 1A; Tao et al., 2019). LEC2 and FUS3 are B3 domain transcription factors that are in the same subfamily as VAL1 and VAL2, proteins that recruit PRC2 to *FLC* chromatin during vernalization (Yuan et al., 2016). In *lec2* or *fus3* single mutants, *FLC::GUS* is re-activated in the zygote, just as it is in wild type plants, but *FLC::GUS* expression in some embryos declined from 2 DAP (*lec*2) and 3 DAP (*fus*3; Table 1B). Binding of LEC2 and FUS3 to the CME within *FLC* chromatin is facilitated by LEC1. As enrichment of first LEC2 and then FUS3 increases from 2 to 6 DAP, there is a corresponding decrease in enrichment of VAL1 at the CME, suggesting that LEC2 and FUS3 antagonize VAL1 binding at the CME during embryogenesis to ensure that *FLC* transcription is maintained following activation by LEC1 (Tao et al., 2019). LEC2 and FUS3 interact with FRI and are required for the recruitment of FRI to the CME and adjacent regions of the *FLC* locus, consistent with the finding that FRI promotes *FLC* expression from 3 DAP. While LEC2 and FUS3 play an important role in maintaining *FLC* expression during early embryogenesis, these proteins are not expressed in post-embryonic stages of *Arabidopsis* allowing VAL1/2 to bind the CME and mediate *FLC* repression during vernalization.

## What Do We Know About Resetting of the Vernalized State?

The resetting of epigenetic regulation in plants varies with the epigenetic modifier involved (Gehring, 2019); for example, there is little evidence to support widespread erasure and replacement of DNA methylation in the developing plant embryo, as occurs in mammalian embryos, because epialleles can be stably inherited between plant generations (Becker et al., 2011; Schmitz et al., 2011). In contrast, the vernalized state, which is mediated by changes in histone modifications at *FLC* chromatin, is reset in each generation (Sheldon et al., 2000). Resetting of *FLC* expression in male and female gametes, the zygote, and developing embryo was examined using an *FLC::GUS* reporter construct (Sheldon et al., 2008; Choi et al., 2009).

Although some GUS activity can be detected in the developing anther, there is no GUS activity in mature pollen or the female gametophyte of vernalized plants (Sheldon et al., 2008; Choi et al., 2009). The timing of *FLC::GUS* reactivation differs between the paternally and maternally inherited transgene. The paternally derived *FLC::GUS* is expressed in up to 50% single-celled zygotes and expression continues throughout embryo development. When inherited from the vernalized maternal parent, expression of *FLC::GUS* is not detected until about 3 DAP (Sheldon et al., 2008). Neither maternally nor paternally inherited *FLC:GUS* are expressed in the fertilized central cell or the developing endosperm (Sheldon et al., 2008).

A mutagenesis screen for resetting mutants identified a hypomorphic mutation in *EARLY FLOWERING 6* (*ELF6*) that impairs resetting of the vernalized state and *FLC* expression in the progeny of vernalized *elf6-5* plants (Crevillen et al., 2014). ELF6 is a jumonji-domain-containing protein that demethylates di- and tri-methylated H3K27, and consistent with this, there is a small increase in H3K27me3 in some regions of *FLC* chromatin associated with somewhat lower *FLC* expression in non-vernalized *elf6-5* plants compared to wild type. Curiously, a null mutant, *elf6-3*, which also has reduced *FLC* expression prior to vernalization, shows no effect on resetting (Crevillen et al., 2014; Tao et al., 2017). A second weak mutant of ELF6, *elf6-4*, also has no effect on the resetting of *FLC* in progeny of a vernalized plant (Tao et al., 2017). Taken together, these observations suggest that ELF6 plays at most a minor role in reactivating *FLC* expression to pre-vernalized levels.

LEC1, LEC2, and FUS3 are essential for resetting expression of *FLC* in the progeny of vernalized plants just as they are for activation of *FLC* in the embryos of non-vernalized plants (Figure 1A; Tao et al., 2017, 2019). Consistent with this, the timing of *FLC::GUS* expression is similar in embryos of both vernalized and non-vernalized plants (Tao et al., 2017).

## Discussion

Our comparison of the genetic requirements for resetting of *FLC* in the progeny of a vernalized plant and those associated with the activation of *FLC* in the young embryo of non-vernalized parents indicates that there is essentially no difference between these processes (Figure 1A). This may seem surprising given that repression of *FLC* during vernalization causes the depletion of active chromatin marks followed by an enrichment with the repressive chromatin modification H3K27me3 across the entire *FLC* locus. We suggest that changes in the nucleosome composition of chromatin that occur during gametogenesis could account in part for these findings (Figures 1A,B).

Firstly, the chromatin of mature sperm cells differs from that in somatic tissue as sperm chromatin lacks histone H3.1, the H3 isoform associated with H3K27me3 (Figure 1A). Instead, sperm chromatin is enriched in H3.10, encoded by the sperm-specific gene *HTR10*, and an H3.3 isoform encoded by *HTR5* (Okada et al., 2005; Rotman et al., 2005; Brownfield et al., 2009; Ingouff et al., 2010; Borg et al., 2011). It has recently been shown that amino acid substitutions around the critical K27 residue in H3.10 (Figure 1B) prevent trimethylation of this residue by PRC2 (Borg et al., 2020). Indeed, H3K27me3 is barely detectable in sperm chromatin and, consistent with this, components of PRC2 are not expressed in *Arabidopsis* sperm cells (Borg et al., 2020). Similarly in monocots, H3K27me3 is observed only in the chromatin of the vegetative cell but not of sperm cells (Sano and Tanaka, 2010; Houben et al., 2011; Pandey et al., 2013). This suggests that the chromatin associated with *FLC* loci, inherited through the paternal gamete of a vernalized plant, would have been stripped of the repressive H3K27me3 mark prior to fertilization. It is hardly surprising then that an *ELF6* null mutant has little effect on resetting (Crevillen et al., 2014; Tao et al., 2017).

Secondly, chromatin dynamics during the development of the mature egg cell are extremely complex and include waves of depletion and presumptive restoration of H3K27m3 (as judged by chromatin localization of LHP1; Baroux and Autran, 2015). Like the sperm cell nuclei, the egg nucleus has a novel complement of histone variants, expressing only *HTR5*, an isoform of H3.3 (Figures 1A,B). Recent data suggest that the vernalized state is transmitted through egg cell chromatin and that the vernalized state is not erased immediately following fertilization (Luo et al., 2020); this is consistent with the observation that *FLC::GUS* inherited from a vernalized maternal parent is not detected until about 3 DAP (Sheldon et al., 2008).

Finally, after fertilization, the paternally inherited H3.10 and the maternally inherited H3.3 are actively removed from chromatin in the zygote but the somatic complement of histone variants is not restored until after *de novo* synthesis of H3.1 and H3.3 during the first zygotic mitosis (Ingouff et al., 2007, 2010). It seems likely then that resetting is merely a consequence of the normal processes by which chromatin is remodeled during gametogenesis and following fertilization.

In conclusion, we suggest that the steps required to activate *FLC* expression in the young embryo are nearly identical regardless of whether the embryo is the progeny of a vernalized or non-vernalized plant. In each case, the chromatin associated with *FLC* loci undergoes major reprogramming during male and female gametogenesis. A shared route to *FLC* activation (Figure 1A) is supported by genetic data showing that the pioneer transcription factor NF-Y and the SWIR chromatin remodeling complex that deposits H2A.Z into chromatin are essential for activating *FLC* expression (Choi et al., 2011; Tao et al., 2017), with LEC2, FUS3, and FRI^c^ being required to fully activate *FLC* expression beyond the day after fertilization (Choi et al., 2009; Tao et al., 2019).

## Author Contributions

EF developed the concept and wrote the manuscript. MR and CH edited the manuscript and designed the figures. All authors contributed to the article and approved the submitted version.

### Conflict of Interest

The authors declare that the research was conducted in the absence of any commercial or financial relationships that could be construed as a potential conflict of interest.
